# Dynamics of microbiota during mechanical ventilation in aspiration pneumonia

**DOI:** 10.1186/s12890-019-1021-5

**Published:** 2019-12-23

**Authors:** Ken Otsuji, Kazumasa Fukuda, Midori Ogawa, Yoshihisa Fujino, Masayuki Kamochi, Mitsumasa Saito

**Affiliations:** 1 Department of Microbiology, School of Medicine, University of Occupational and Environmental Health, Japan, Kitakyushu, Japan; 2 Department of Intensive Care Medicine, Hospital of the University of Occupational and Environmental Health, Japan, Kitakyushu, Japan; 3 Department of Environmental Epidemiology, Institute of Industrial Ecological Sciences, University of Occupational and Environmental Health, Japan, Kitakyushu, Japan

**Keywords:** Aspiration pneumonia, Microbiota, Anaerobes, Mechanical ventilation, Dynamics

## Abstract

**Background:**

The emergence of multi-drug resistant pathogens is an urgent health-related problem, and the appropriate use of antibiotics is imperative. It is often difficult to identify the causative bacteria in patients with aspiration pneumonia because tracheal aspirate contains contaminants of oral bacteria. We investigated the dynamics of microbiota in mechanically ventilated patients with aspiration pneumonia to develop a treatment strategy.

**Methods:**

Twenty-two intubated patients with aspiration pneumonia were recruited. Saliva and tracheal aspirate of the subjects were collected at three time points: (A) within 2 h after intubation, (B) just before administration of antibiotics, and (C) 48-72 h after administration of antibiotics. The microbiota in each specimen was analyzed by using the 16S rRNA gene clone library sequencing method. Bacterial floras of the samples were analyzed by principal component analysis.

**Results:**

Principal component analysis based on the composition of genus revealed that although the changes of microbiota in the saliva from (A) to (B) were not clear, the composition of anaerobes in the tracheal aspirate (B) was lower than (A). In fact, the reduction of anaerobes, not in the saliva but in the tracheal aspirate from (A) to (B), was confirmed by incident rate ratios estimated by a multilevel Poisson regression model (*p* < 0.001). The extent of decrease in anaerobes was fully dependent on the time difference between the sampling of tracheal aspirate (A) and (B)—in particular, over 3 h of mechanical ventilation. This indicates that the alterations of microbiota (involving the reduction of anaerobes in the lower respiratory tract) occurred during mechanical ventilation prior to the administration of antibiotics. After the administration of antibiotics, *Enterobacter* spp., *Corynebacterium* spp., *Pseudomonas aeruginosa*, *Klebsiella pneumoniae*, *Staphylococcus aureus*, and *Granulicatera adiacens* were predominantly detected in the tracheal aspirate (C).

**Conclusion:**

The microbiota of the lower respiratory tract changes dynamically during mechanical ventilation and during the administration of antibiotics in intubated patients with aspiration pneumonia. Antibiotics should be selected on the premise that dynamic changes in microbiota (involved in the reduction of anaerobes) may occur during the mechanical ventilation in these patients.

## Background

Aspiration pneumonia is an infectious process caused by the inhalation of oropharyngeal secretions in which pathogenic bacteria are colonized [1]. Several studies show that aspiration is implicated in 5 to 20% of community acquired pneumonia [2–4] and 15 to 75% of healthcare-associated pneumonia [5–7]. As for causative agents, anaerobic bacteria and facultative anaerobic bacteria such as *Staphylococcus aureus*, *Klebsiella pneumoniae,* and *Escherichia coli* were detected from patients with aspiration pneumonia [1, 8–10]. Culture methods are typically used to identify the causative agents [11]. However, it is often difficult to identify the causative bacteria in patients’ tracheal aspirate via the culture method because anaerobic bacteria are usually difficult to culture, and because tracheal aspirate contains contaminants of indigenous oral bacteria. Although over 700 bacterial species/phylotypes have been detected in the oral cavity by genetic analysis, a large number of anaerobic species have yet to be detected by culture using conventional methodologies [12, 13]. Therefore, it is difficult to evaluate pathogens of aspiration pneumonia via culture-dependent methods. Difficulty in identification of causative bacteria may lead to inappropriate administration of antibiotics and the emergence of drug-resistant pathogens. The proportion of nosocomial infections caused by multi-drug resistant pathogens is increasing, resulting in an increased length of hospital stay, mortality, and cost [14]. The emergence of multi-drug resistant pathogens is an urgent health-related problem, and proper use of antibiotics is necessary.

Advances in DNA sequencing technologies have allowed analysis of diverse microbiota and the evaluation of pathogenic bacteria by using culture-independent methods [15–19]. In cases of aspiration pneumonia, 16S rRNA gene sequencing is used for the identification of causative bacteria [20–22]. However, how microbiota of the oral cavity and lower respiratory tract changes as a result of antibiotic administration or mechanical ventilation has not been sufficiently evaluated. In particular, high concentrations of oxygen may be administered during mechanical ventilation, and we have found no reports regarding the dynamics of anaerobes in the lower respiratory tract during mechanical ventilation. We therefore hypothesized that microbiota in patients with aspiration pneumonia—especially with anaerobes in the lower respiratory tract—change dynamically during mechanical ventilation and the administration of antibiotics. Recognition of the actual causative agents using 16S rRNA gene sequencing for microbiota analysis may lead to new treatment procedures for pneumonia. To confirm our hypothesis, we evaluated the changes in the microbiota of oral cavities and lower respiratory tracts in cases of mechanically ventilated patients with aspiration pneumonia using the clone library analysis (based on highly accurate 16S rRNA gene sequencing).

## Methods

### Subject enrollment

This is a single center prospective study undertaken at the hospital of the University of Occupational and Environmental Health in Japan between March 2016 and March 2018. Inclusion criteria and exclusion criteria of the subjects are as follows.

#### Inclusion criteria

- Intubated (endotracheal) due to diagnoses of respiratory failure accompanying aspiration (considerations were background information such as medical history inclusive of status of comatose).
- 18 years of age or older.
- Did not receive antibiotics within the past 2 weeks.

#### Exclusion criteria

- Not diagnosed with pneumonia according to the IDSA/ATS (Infectious Diseases Society of America/American Thoracic Society) guidelines [11].
- Negative result for PCR.

Antibiotics were administered at the decision of the clinician: considerations were fever, elevated inflammatory response in laboratory data, properties of tracheal aspirate, and appearance of pneumonia on the image (chest X-ray or computed tomography).

### Sample collection

The saliva and tracheal aspirate specimens of the subjects were collected aseptically at the following three time points: (A) within 2 h after intubation, (B) just before administration of antibiotics, (C) 48-72 h after the administration of antibiotics. Each specimen was termed as saliva (A), (B), (C) and tracheal aspirate (A), (B), (C), respectively. The saliva was aspirated through suction tubes from the oral cavity and the tracheal aspirate was collected using suction catheters through endotracheal tubes from the lower respiratory tract. The tracheal aspirate (B) were cultured under microaerophilic conditions at 35 °C with 5% CO_2_ on standard microbiological media in a clinical laboratory of the hospital. To reduce the potential effects of oral care, the samples were collected 2 h before or after the scheduled oral care. After collecting these samples, they were stored immediately at 4 °C until analyzed (within 7 days). Clinical data were extracted from the electronic medical records. Tracheal diameters were evaluated at the level of the sternoclavicular joint with computed tomography images. This study was approved by the Ethics Committee of Medical Research, University of Occupational and Environmental Health, Japan (No. H27–233).

### 16S rRNA gene sequencing analysis

The total bacterial cell count in the samples were confirmed by the epifluorescence staining method described previously [23, 24]. Bacterial DNA were extracted with sonication method and 16S rRNA genes were amplified with a universal primer set [25] described previously [23, 24]. The PCR products were cloned into *Escherichia coli* using a TOPO TA cloning kit and a total of 96 colonies were randomly selected for sequencing analysis. Data analysis was performed using ‘Sequencing Analysis V.5.2’ (Applied Biosystems). Additional details of the method are described in Additional file 1: Appendix S1. The highly accurate sequences were classified into the level of genus with RDP (ribosomal database project) classifier (confidence threshold 90%) [26]. The sequences of less than 90% confidence threshold value were deemed unclassified. Genera with proportions less than 0.1% of the total number of clones were titled ‘others’. Moreover, the in-house database (Additional file 2: Appendix S2) containing 5878 16S rRNA gene sequences of type strains using the BLAST (basic local alignment search tool) algorithm was used for more detailed analysis at the level of species. All sequences were submitted to public database (DNA data bank of Japan, Accession number: LC350341-LC360093).

### Statistics

To compare the microbiota, principal component analysis (PCA) was performed based on the proportion of the genus. In order to visualize the complicated data, PC1 and PC2 were selected for a two dimensional PCA map. R software version 3.4.0. (http://cran.r-project.org/) was used for the PCA as described previously [27]. To confirm the changes of the anaerobes in the saliva and tracheal aspirate during mechanical ventilation, incident rate was defined as the number of clones of anaerobic bacteria per sample. Bergey’s Manual of Systematics of Archaea and Bacteria [28] was referenced for obligate anaerobes, aerobes, and facultative anaerobes. Incident rate ratios (IRR) were estimated by a multilevel Poisson regression model in which retrieved samples from the same individual were nested, considering for multiple samples from same individual. STATA14 was used for statistical analysis with a multilevel Poisson regression model. The change in total bacterial count between tracheal aspirates (A) and (B) were assessed using a paired t-test. *P* < 0.05 was considered statistically significant.

## Results

### Subject characteristics

**Fig. 1:**
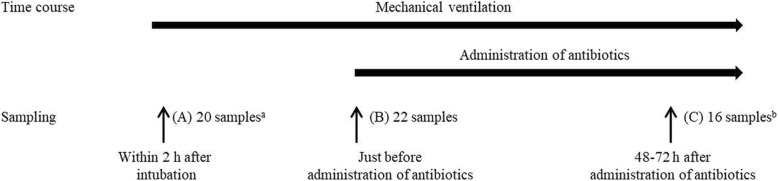
Trial profile. ^a^Two cases were failure to obtain samples. ^b^Six cases were negative result for PCR or extubated at (C)

A total of 24 subjects met the inclusion criteria. However, two subjects were excluded from the study: one did not result in diagnosis of pneumonia, and the other had a negative result for PCR of the saliva and tracheal aspirate at each point because of Mendelson’s syndrome. The remaining 22 subjects were analyzed. The subjects were hospitalized with various primary diagnoses [examples: comatose due to cerebral hemorrhage, drug overdose, and cardiopulmonary arrest] (Table 1). The time duration from collecting samples (A) to (B) ranged from 1.0–74.0 h (mean 10.3 h). There were two cases (case 18, 21) where the samples failed to be collected within 2 h after intubation, and there were six cases (case 7, 10, 11, 13, 15, 17) from which samples (C) could not be collected because of extubation or negative PCR results (Fig. 1).

**Table 1:** Characteristics of the subjects

Subject	Sex	Primary diagnosis^a^	Co-morbidities	Interval I to (A) (h)^b^	Interval (A) to (B) (h)^c^	TD (mm)	TD (long/short)^d^	FIO_2_ (%)	Medication		Days				Mortality^e^
									Vasopressor	Immuno-modulator	Admission to recruitment	ICU stay	Mechanical ventilation	Hospital stay	
1	M	Aspiration pneumoniae	CVD, COPD	1	11	29 × 24	1.20	30–50	+	–	33	5	9	76	survive
2	M	Asphyxia	Thyroid cancer, HT	1	2	22 × 18	1.22	35–50	–	–	6	6	5	26	survive
3	F	Drug overdose	Depression	0	3	21 × 18	1.16	30–40	+	–	0	5	4	12	survive
4	M	CPA	DCM, CRF	1	15	23 × 19	1.21	45–60	+	–	0	9	14	14	dead
5	M	Epidural hematoma	DM, HT, LC	1	11	32 × 19	1.68	35–45	–	–	3	11	8	25	survive
6	M	Subarachnoid hemorrhage	–	0.5	12	24 × 20	1.20	35–50	–	–	0	4	4	30	survive
7	M	Drug overdose	Depression	0.5	4	23 × 23	1.00	21–50	–	–	0	2	2	4	survive
8	F	Cerebral hemorrhage	SLE, DM, HT	0.5	74	21 × 20	1.05	30–40	–	–	1	8	23	24	survive
9	M	Heart failure	HF, DM, HT, CKD	0.5	1	22 × 21	1.04	45–80	–	–	0	45	44	50	dead
10	F	Convulsion	Epilepsy	1	3	22 × 20	1.10	25–30	–	–	0	4	3	10	survive
11	M	Convulsion	Epilepsy	1.5	2	22 × 20	1.10	25–30	–	–	0	2	2	16	survive
12	M	Aspiration pneumoniae	COPD	0.5	1	20 × 19	1.05	50–60	–	–	1	15	36	37	survive
13	F	Subarachnoid hemorrhage	–	0.5	11	NA	NA	30	–	–	0	4	2	36	survive
14	F	CPA	HF, CVD	1	3	20 × 19	1.05	35–70	–	–	0	8	12	12	survive
15	F	Cerebral hemorrhage	HT	0.5	1	19 × 18	1.05	35	+	–	0	7	6	55	survive
16	M	Subarachnoid hemorrhage	SAS	0.5	3	19 × 19	1.00	35–90	–	–	0	30	51	96	survive
17	M	CPA	CVD, HF, HT	1	12	26 × 21	1.23	35–50	+	–	0	7	11	11	survive
18	F	Aortic dissection	DM, HT	NA	NA	24 × 17	1.41	45–70	–	–	4	16	16	16	dead
19	M	Aspiration pneumoniae	CVD	2	23	25 × 24	1.04	30–40	–	–	3	25	96	99	survive
20	F	Aspiration pneumoniae	Brainstem dysfunction	1	3	20 × 12	1.66	30–45	–	–	5	23	117	120	survive
21	M	Aspiration pneumoniae	HF, DM, HT	NA	NA	26 × 23	1.13	45–95	+	–	0	13	13	13	survive
22	F	Convulsion	Epilepsy, DM	0.5	11	22 × 18	1.22	40–50	+	–	0	7	7	29	survive

*M* male, *F* female, *CPA* cardiopulmonary arrest, *CVD* cerebral vascular disease, *COPD* chronic obstructive pulmonary disease, *HT* hypertension, *DCM* dilated cardiomyopathy, *CRF* chronic renal failure, *DM* diabetes mellitus, *LC* liver cirrhosis, *SLE* systemic lupus erythematosus, *HF* heart failure, *CKD* chronic kidney disease, *SAS* sleep apnea syndrome. ^a^Diagnosis resulted in respiratory failure, ^b^Interval I to (A) = time from tracheal intubation to sample collection (A), ^c^Interval (A) to (B) = interval of collecting sample (A) and (B), (A) = samples collected within 2 h after intubation, (B) = samples collected just before administration of antibiotics, TD = tracheal diameter, ^d^TD (long/short) = the ratio of longest/shortest tracheal diameter. ^e^None of the cases were died of aspiration pneumonia

### Microbiota of saliva and tracheal aspirate

A total of 116 samples of saliva and tracheal aspirate from 22 subjects were analyzed. In this study, 9753 of the partial 16S rRNA gene sequences were analyzed. RDP classifier analysis resulted in a total 118 genera—including 76 genera that are defined as ‘others’. The compositions of clones assigned to each genus detected in the saliva and tracheal aspirate specimens are shown in Additional file 3 (Figure S1–1, S1–2). The results of unsupervised hierarchical clustering of genus for samples of saliva and tracheal aspirates are shown in Additional file 4 (Additional file 4: Figure S2–1, S2–2). As a result of unsupervised hierarchical clustering, the relationship between the detected phylotypes and the timing of recruitment to the study or the timing of specimen collection was not clear. The top three predominant genera detected in the saliva and the tracheal aspirate at each period are shown in Additional file 5 (Table S1). *Streptococcus* was predominantly detected in each period of both saliva and tracheal aspirate.

### Culture vs clone library method

The culture results and phylotypes detected by clone library method in each case are shown in Table 2. Although the culture method detected predominant phylotypes of the tracheal aspirate (B) in many [85.7% (18/21)] of the subjects, it failed to detect any in the remaining cases (case 9, 14 and 17). After the administration of antibiotics, *Enterobacter* spp. [*asbriae* (99%), *cloacae* (98%)]*, Corynebacterium* spp. [*propincuum* (99%), *acoolens* (99%)]*, Pseudomonas aeruginosa* (99%)*, Klebsiella pneumoniae* (99%)*, Staphylococcus aureus* (99%), and *Granulicatera adiacens* (99%) were predominantly detected in the tracheal aspirate (C) (% indicates similarities with the type strain). There were no cases in which the predominant phylotype of the tracheal aspirate was an anaerobe in the results of clone library analyses.

**Table 2:** Comparison between culture results and predominant phylotypes detected by clone library method of tracheal aspirate in each subjects

Case	Culture Result at (B)^a^	Clone Library Analysis of 16S rRNA gene			Antibacterial Agent
		Predominant Phylotype, Proportion (%)			
		(A)	(B)	(C)	
Culture result detected predominant phylotype (B)					
1	***Klebsiella pneumoniae*** **+++**, *Staphylococcus aureus* +, Oral flora^b^	*Lactobacillus,* 50	***Klebsiella,*** **56**	*Lactobacillus,* 36	Piperacillin-Tazobactam
2	***Streptococcus agalactiae*** **+++**, **Oral flora**	*Streptococcus,* 84	***Streptococcus,*** **94**	*Streptococcus,* 42	Meropenem
3	***Raoultella planticola*** **+++**, Oral flora, *Staphylococcus aureus* +++, Yeast +++	*Streptococcus,* 64	***Raoultella*, 82**	*Enterobacter,* 98	Ampicillin-Sulbactam
4	***Streptococcus salivarius***	***Streptococcus,*** **74**	*Pseudomonas,* 84	*Streptococcus,* 58	Ampicillin-Sulbactam
5	**Oral flora**	*Neisseria,* 73	***Neisseria,*** **58**	*Neisseria,* 58	Ampicillin-Sulbactam
6	***Streptococcus mitis***	*Streptococcus,* 76	***Streptococcus,*** **95**	*Corynebacterium,* 40	Ampicillin-Sulbactam
7	***Streptococcus pneumoniae*** **++**, **Oral flora,** *Staphylococcus aureus* < +	*Lactobacillus,* 47	***Streptococcus,*** **88**	NA	Ampicillin-Sulbactam
8	***Klebsiella pneumoniae*** **++**, Oral flora, Yeast ++	*Streptococcus,* 75	***Klebsiella,*** **64**	*Enterobacter,* 58	Ampicillin-Sulbactam
10	**Oral flora**	*Streptococcus,* 67	***Streptococcus,*** **77**	NA	Ampicillin-Sulbactam
11	**Oral flora**	*Streptococcus,* 82	***Streptococcus,*** **84**	NA	Ampicillin-Sulbactam
12	**Oral flora**	*Streptococcus,* 69	***Streptococcus,*** **52**	*Neisseria,* 30	Meropenem
15	***Enterobacter aerogenes*** **+++**, Oral flora	*Streptococcus,* 85	***Enterobacter,*** **76**	NA	Ampicillin-Sulbactam
16	*Staphylococcus aureus* +, **Oral flora**	*Streptococcus,* 42	***Streptococcus,*** **93**	*Klebsiella,* 100	Ampicillin-Sulbactam
18	***Staphylococcus aureus*** **+++**, *Klebsiella pneumoniae* +++, Oral flora	NA	***Staphylococcus,*** **73**	*Staphylococcus,* 60	Ampicillin-Sulbactam
*Enterobacter cloacae complex* ++,					
19	***Klebsiella pneumoniae*** **+++,** *Staphylococcus aureus* +++, *Pseudomonas aeruginosa* +++, Oral flora	*Pseudomonas,* 30	***Klebsiella,*** **78**	*Staphylococcus,* 29	Ampicillin-Sulbactam
20	***Haemophilus parainfluenzae*** **+**, Oral flora	*Haemophilus,* 60	***Haemophilus,*** **90**	*Granulicatella*, 41	Ampicillin-Sulbactam
21	*Escherichia coli *+++, **Oral flora,** *Corynebacterium* sp. +++, Yeast +	NA	***Lactobacillus,*** **70**	*Pseudomonas,* 55	Ampicillin-Sulbactam
22	***Streptococcus*** **sp ++**, **Oral flora**	*Streptococcus,* 50	***Streptococcus,*** **47**	*Streptococcus,* 48	Ampicillin-Sulbactam
Culture result could not detect predominant phylotype (B)					
9	GPR (not detectable) +++, *Leuconostoc pseudomesenteroides* ++	*Lactobacillus,* 68	*Streptococcus,* 87	*Pseudomonas,* 98	Ampicillin-Sulbactam
14	No growth	*Leuconostoc,* 54	*Streptococcus,* 90	*Streptococcus,* 69	Ampicillin-Sulbactam
17	*Klebsiella pneumoniae* ++	*Granulicatella,* 44	*Granulicatella*, 37	NA	–
Not analyzed					
13	NA	*Streptococcus,* 55	*Streptococcus,* 78	NA	–

*NA* not analyzed, (A) = samples collected within 2 h after intubation, (B) = samples collected just before administration of antibiotics, (C) = samples collected 48–72 h after the administration of antibiotics, Proportion = the ratio of clones of predominant phylotype to total clone. Matched result of culture result and predominant phylotype (B) are indicated as bold. Predominant phylotype detected after the administration of antibiotics are indicated with underline. ^a^Culture results of case 4 and 9 are at (A). ^b^Oral flora include *Streptococcus*, *Neisseria* and *Lactobacillus*

### Principal component analysis

**Fig. 2:**
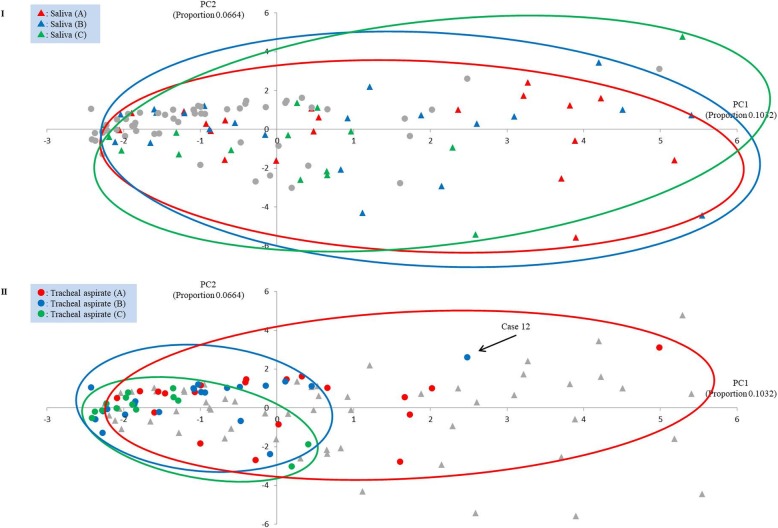
The change of microbiota in saliva (I) and tracheal aspirate (II). Gray circle indicates the tracheal aspirate and gray triangle indicates the saliva. PC1: Principal Component 1, PC2: Principal Component 2, (A): samples collected within 2 h after intubation, (B): samples collected just before administration of antibiotics, (C): samples collected 48–72 h after the administration of antibiotics

The results of PCA based on the detected phylotypes at the genus level are shown in Fig. 2. The changes in the saliva from (A) to (B) were not clear. On the other hand, in the tracheal aspirates (A and B), only (B) tended to cluster into the negative side of PC1. The compositions of anaerobic bacteria occupied the major positive eigenvectors of PC1 (see Additional file 6: Figure S3). The changing location of spots on the PCA suggests that the composition of anaerobes in tracheal aspirate (B) was lower than (A).

### Proportion of anaerobes

**Fig. 3:**
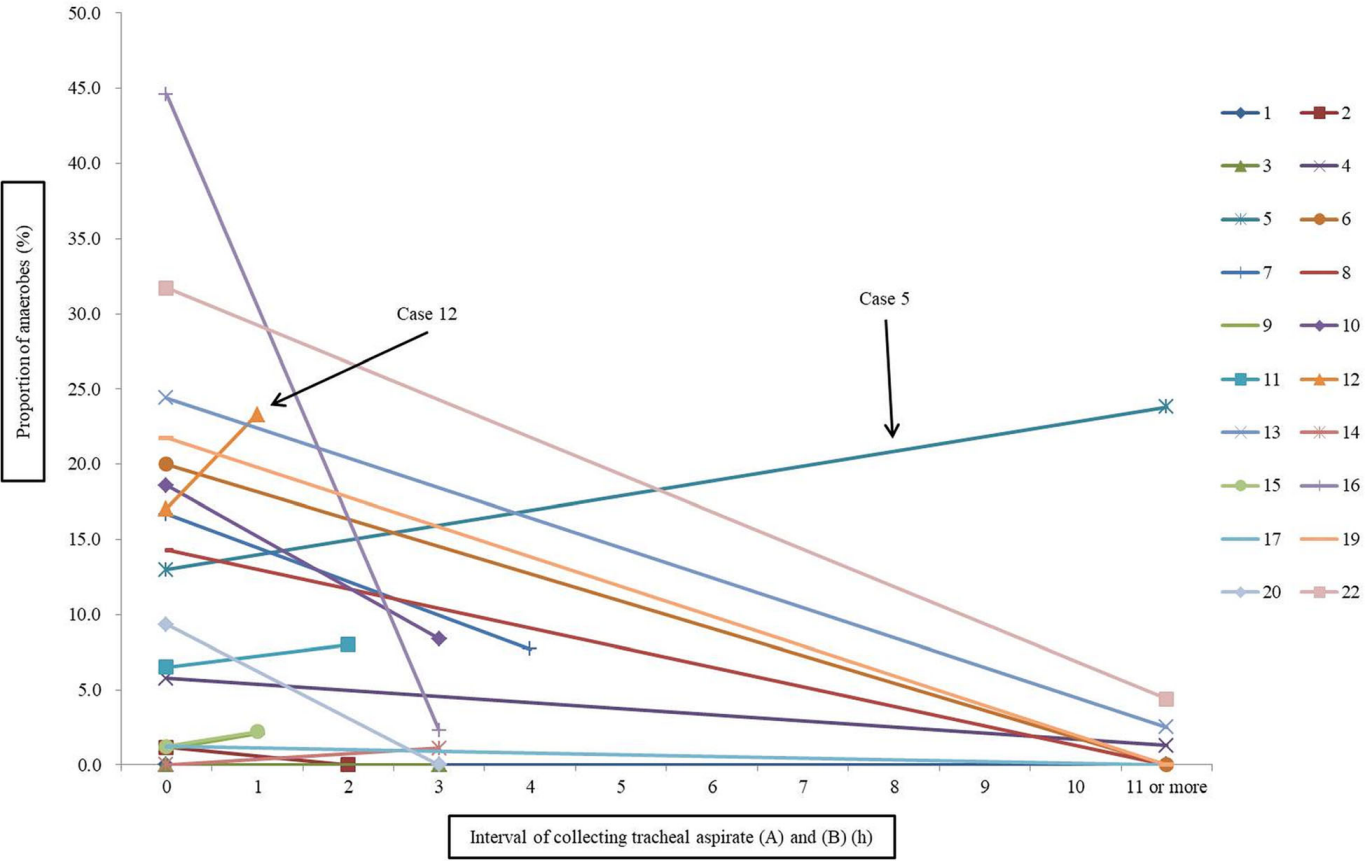
Changes in proportion of anaerobes in tracheal aspirate from (A) to (B) during mechanical ventilation (individual cases). Proportion of anaerobes at “0” indicates proportion of anaerobes in tracheal aspirate (A), (A): samples collected within 2 h after intubation, (B): samples collected just before administration of antibiotics

Dynamics of microbiota in saliva and tracheal aspirate is shown in Additional file 7 (Figure S4). The proportions of anaerobes at each period in saliva samples were (A); 25.5% (421/1650), (B); 24.7% (456/1843), and (C); 16.6%(227/1366), and in tracheal aspirate samples were (A); 12.3% (206/1678), (B); 3.9% (74/1880), and (C); 2.6% (35/1336). There was no significant difference in the proportion of anaerobes between saliva (A) to (B) (IRR: 0.97, 95% CI 0.85–1.12, *p* = 0.76). On the other hand, the proportion of anaerobes were significantly lowered between tracheal aspirate (A) to (B)(IRR: 0.34, 95% CI 0.26–0.45, *p* < 0.001). Figure 3 shows the changes in proportion of anaerobes in tracheal aspirate from (A) to (B) during mechanical ventilation (individual cases). As a result of multivariate analysis using clinical factors such as FiO_2_, tracheal diameter, vasopressor use, ICU stay, mechanical ventilation days, hospital stay and mortality, there was no significant relationship between the proportion of anaerobes and these clinical factors. However, the extent of decrease in anaerobes was fully dependent on the time difference between the sampling of tracheal aspirate (A) and (B). There was no significant decrease of anaerobes in the tracheal aspirate (B) collected within 2 h after collecting the tracheal aspirate (A) (IRR: 1.23, 95% CI 0.73–2.05, *p* = 0.43); a significant decrease in anaerobes occurred in tracheal aspirate (B) collected 3 to 10 h after collecting tracheal aspirate (A) (IRR: 0.22, 95% CI 0.13–0.38, *p* < 0.001) and collected 11 h or longer after collecting tracheal aspirate (A) (IRR: 0.23, 95% CI 0.15–0.35, *p* < 0.001). It is noteworthy that most anaerobes were not detected in tracheal aspirate (B) which was collected 11 h or longer after collection (A) (Fig. 3).

## Discussion

DNA sequencing technologies have recently advanced and have therefore been applied to clinical specimens—although 16S rRNA gene sequencing cannot evaluate certain properties of the detected bacteria (such as drug sensitivity), it is suitable for evaluating bacterial flora and pathogenic bacteria. Because highly diverse microbial communities including anaerobes and indigenous oral bacteria harbor in aspiration pneumonia, 16S rRNA gene sequencing analysis is frequently used to evaluate the causative bacteria [20–22]. The dynamics of the bacterial flora in the lower respiratory tract in mechanically ventilated patients are evaluated as follows [29–31]. Berdal and colleagues [29] evaluated tracheal suction samples from 48 h after intubation to every 72 h thereafter in 74 mechanically ventilated patients. They found the microbiota in the upper and lower airways of mechanically ventilated patients to be closely correlated and relatively stable over a period of 72 h. Kelly and colleagues [30] evaluated oropharyngeal and endotracheal secretions sampled within 24 h of intubation, and every 48–72 h thereafter, from 15 mechanically ventilated patients with respiratory failure which were compared with samples obtained from a healthy control group. They found that critically ill subjects had lower initial diversity in upper and lower respiratory tract microbiota compared to those of the healthy control. And, the diversity further diminished over time on the ventilator. Zakharkina and colleagues [31] evaluated endotracheal aspirate samples collected at the time of intensive care unit admission, and then subsequently twice a week from 35 mechanically ventilated patients for a non-infectious cause of respiratory failure. They found that mechanical ventilation—but not antibiotic administration—was associated with changes in the respiratory microbiome, and that dysbiosis of microbial flora in the respiratory tract was most profound in patients whom developed ventilator associated pneumonia (VAP). However, we found no reports evaluating the dynamics of microbiota immediately after aspiration regarding mechanically ventilated patients with aspiration pneumonia.

In this study, a total of 116 samples of saliva and tracheal aspirate from 22 mechanically ventilated patients with aspiration pneumonia were collected. We analyzed the changes in the microbiota during mechanical ventilation, and during antibiotic treatments. To our knowledge, this is the first report suggesting that microbiota in the lower respiratory tract may change dynamically during mechanical ventilation *before administration of antibiotics* in intubated patients with aspiration pneumonia. It is worthy of note that in the tracheal aspirate from (A) to (B), the proportion of anaerobes decreased significantly, and that some facultative anaerobes such as *Staphylococcus, Klebsiella, and Enterobacter* tended to increase (Additional file 7: Figure S4). Because the bacterial count (Additional file 3: Figure S1–2) in tracheal aspirate (B) (mean 1.00 × 10^8^ cells/mL) was significantly lower than those of the tracheal aspirate (A) (mean 1.07 × 10^9^ cells/mL) (t = 2.52, df = 19, *p* = 0.02), it is not conclusive that the number of bacteria of these facultative anaerobes has actually increased; caution is necessary for this interpretation. Although we cannot deny the possibility that other factors related to the subjects may have had an effect on the outcome, we can assume that the anaerobes in the tracheal aspirate were lowered mainly due to the effect of mechanical ventilation. Notable fluctuations of microbiota in the tracheal aspirate were significantly dependent on the mechanical ventilation times—in particular when it was over 3 h. Although further investigation is necessary to explain this phenomenon clearly, several assumptions can be made. There is a possibility that the normal lung microbiome was disrupted by mechanical ventilation, or that those fluctuations may be caused by oxygen concentration increase via mechanical ventilation (because obligate anaerobes cannot survive in the presence of oxygen). This phenomenon supports previous studies regarding anaerobic organisms as major pathogens in non-intubated patients with aspiration pneumonia [1, 8–10]. However, they are rarely identified in intubated patients with aspiration pneumonia [10, 32].

There were two exception cases (case 5 and 12). In case 12, the duration of mechanical ventilation treatment (only 1 h) may not have been long enough to significantly affect the microbial flora (Fig. 2). Regarding case 5, the interval between collecting tracheal aspirate (A) and (B) was 11 h. However, the anaerobes did not decrease (Fig. 3). The longest diameter and long/short of the tracheal diameter of this case were largest (longest TD: 32 mm, mean 22.9; long/short of TD: 1.68, mean 1.16) among the subjects as described in the results (Table 1); it is possible that the cuff of the endotracheal tube was not adequately adhered to the trachea. Therefore, oropharyngeal secretions may have flowed into the lower respiratory tract frequently (or perhaps constantly). Since VAP is considered to be caused by an inflow of oropharyngeal secretions into the lower respiratory tract [33, 34], perhaps it should be recognized that a large tracheal diameter can be a substantial risk. The fraction of inspiratory oxygen administered to the subjects was 21–50% to 30–95% (Table 1). In case 13, the anaerobes were markedly reduced. However, in this case, the patient was administered a relatively low fraction of inspiratory oxygen (FIO_2_: 30%); therefore, it may be deduced that a high concentration of oxygen is not always necessary in decreasing anaerobes in the lower respiratory tract.

In this study, the culture method detected predominant phylotypes of the tracheal aspirate (B) in many of the cases (Table 2). However, the culture method could not detect predominant phylotypes in three of the cases (case 9, 14 and 17). In case 9, since *Lactobacillus* is a gram positive rod, it is possible that *Lactobacillus* was detected in the culture but it could not be identified in the clinical laboratory of the hospital. In case 17, *Granulicatella* was difficult to culture because of its pyridoxal dependence and slow growth characteristics [35]; it may not have been detected by the culture method for these reasons. It is not clear why no growth was detected in case 14. After the administration of antibiotics, *Enterobacter asbriae, E. cloacae, Corynebacterium propinquum*, *C*. *acoolens*, *Pseudomonas aeruginosa, Klebsiella pneumoniae, Staphylococcus aureus,* and *Granulicatera adiacens* were predominantly detected in the tracheal aspirate (C) (Table 2). These bacteria mostly correspond with the antibacterial drug resistant bacteria detected in the intensive care unit [36] and the pathogenic bacteria of VAP [34]. It is noteworthy that these pathogenic bacteria were detected as being predominant 48-72 h after the administration of antibiotics. Although antibacterial susceptibilities of causative bacteria—except the isolates—were not evaluated in this study, these bacteria are generally associated with antimicrobial resistance. These bacteria might be major pathogens when initial treatment is ineffective. However, after the administration of antibiotics in this study, the causal relationship between the patient background and the predominantly detected bacteria was not clear. Since most of these bacteria can be detected by culture method, a personalized approach using the culture method is a practical strategy. There were only two cases (13 and 17) where patients were intubated but did not receive antibiotics. Large scale analysis of the dynamics of bacterial flora of such cases may provide interesting results and are expected to be in future research.

From the present findings, it can be deduced that antibiotics should be selected on the premise that dynamic changes in microbiota (involved in the reduction of anaerobes in the lower respiratory tract) may occur during mechanical ventilation in intubated patients with aspiration pneumonia. It was suggested that antibiotic treatment for anaerobes in these cases may not be appropriate. Additional pathogenic investigation should be conducted after over 3 h from initiating mechanical ventilation. However, because this is a single location observational study, and because the sample size was small, evaluation by larger scale interventional analysis is required for finding the most appropriate antibiotic regimen. The use of wide spectrum antibiotics (unnecessarily) leads to the emergence of drug resistant bacteria—which is now an urgent health-related problem. Needless to say, accurate identification of pathogenic bacteria and the justified usage of antibiotics are both imperative.

This study has several limitations. First, the sample size was small, which is one of our major limitations. Because of the small sample size, the full dynamics of the microbiota during mechanical ventilation in aspiration pneumonia could not be evaluated. It is difficult to say that sufficient information has been obtained for treatment strategy from this study. Second, the use of the Sanger sequencing method for analysis of the bacterial 16S rRNA gene is another limitation. NGS (next generation sequencer) is commonly used for analysis of human microbiota [15]. An important advantage of NGS is the abundance of available nucleotide sequence information [37]. The number of clones identified by the Sanger method in this study is 96 at maximum per sample and significantly fewer than NGS; this is the essence of the limitation. However, there are many kinds of *Streptococcus* spp. in the oral and in the respiratory tract, which most NGS cannot classify at the species level [38] because their sequences of 16S rRNA genes are too similar. The Sanger method used in this study is targeting 550 bp of the gene including three variable regions (V3, V4 and V5), so that it is possible to classify bacteria in more detail. Third, background samples were not obtained during sequence analysis in this study. When using sequencing techniques to assess microbiota in low biomass environments, care should be taken regarding the potential contamination of background [39]. In this study, the DNA extract from only PBS (without specimen) was used as the control. No obvious band (with 2% agarose gel electrophoresis analysis) was detected from the control sample after the PCR (30 cycles) and the cloning-sequencing analyses were performed with only PCR positive samples. Since significant amplification was not observed from the control samples and most bacterial phylotypes detected in this study are commonly found in the upper and lower respiratory tract, the contamination of the background was regarded small and insignificant. However, we cannot deny that background contamination may have influenced the results of the analysis in some way. The final limitation to be discussed regarding this study is that samples for DNA analysis were stored at 4 °C until time of analysis. In this study, not only the evaluations of the microbiota but also the number of bacteria contained in the specimen were analyzed. In order to avoid changes of the bacterial count result by repeated freezing and thawing of the samples, the samples were stored not at − 20 °C or − 80 °C but at 4 °C until analysis. We cannot deny the possibility that this method may have affected on the results in some way—however, as a result in this study, the microbiota of the saliva had hardly changed from (A) to (B) and the anaerobic bacteria count significantly decreased in the tracheal aspirate. Therefore, storing the sample at 4 °C seems to have had little to no influence on the results.

## Conclusion

The microbiota of the lower respiratory tract changes dynamically during mechanical ventilation before administration of antibiotics in intubated patients with aspiration pneumonia. The potential of microbiota alteration should be considered for the proper antibiotic treatment in these patients.

## Supplementary information

**Additional file 1: Appendix S1.** Appendix describing additional detail of the method and figure legends of additional figure.

**Additional file 2: Appendix S2.** In-house database containing 5878 16S rDNA sequences of type strains.

**Additional file 3: Figure S1–1, S1–2.** Proportions of bacterial flora in saliva and tracheal aspirate (individual cases).

**Additional file 4: Figure S2–1, S2–2.** Unsupervised hierarchical clustering of genus for samples of saliva and tracheal aspirate.

**Additional file 5:** **Table S1.** Predominant Phylotypes Detected by Clone Library Method in 22 Subjects.

**Additional file 6:** **Figure S3.** Eigenvectors of PC 1 and 2 by bacterial genera.

**Additional file 7:** **Figure S4.** Dynamics of microbiota in saliva and tracheal aspirate.

## Data Availability

Datasets analyzed for this study are available from the corresponding author on reasonable request.

## Abbreviations

IRR: Incident rate ratios
PCA: Principal component analysis
VAP: Ventilator associated pneumonia
