# Supplementary material for: Dynamics of microbiota during mechanical ventilation in aspiration pneumonia
Source: BMC Pulm Med. 2019 Dec 23;19:260. doi: 10.1186/s12890-019-1021-5 (PMC6929358; doi:10.1186/s12890-019-1021-5)
Supplement: Supplementary file 1 — Additional file 1: Appendix S1. Appendix describing additional detail of the method and figure legends of additional figure. [file 12890_2019_1021_MOESM1_ESM.docx]

Dynamics of microbiota during mechanical ventilation in aspiration pneumonia

Ken Otsuji, MD; Kazumasa Fukuda, PhD; Midori Ogawa, PhD; Yoshihisa Fujino, MD, PhD; Masayuki Kamochi, MD, PhD; Mitsumasa Saito MD, PhD

**Appendix S1:** Appendix describing additional detail of the method and figure legends of additional figure.

**Additional detail of the method**

*Total bacterial count*

The collected samples of saliva and tracheal aspirate were vortexed and split for the total bacterial cell count and bacterial DNA extraction. The total bacterial cell count in the samples were confirmed by the epifluorescence staining method described previously [1,2]. The sample dilution (100 µL) was mixed with 900 µL of ethidium bromide solution (100 µg/mL in 0.1 M phosphate pH 8.5 buffer, 5% NaCl, 0.5 mM sodium EDTA) and incubated for 10 min at room temperature. The mixture (1.0 mL) was then filtrated through a pore-filter of 0.2 μm (Millipore, Bedford, MA, U.S.A) and rinsed once with 3.0 mL of sterile distilled water. Bacterial shaped objects on the filter were counted under an Olympus BX40 fluorescence microscope (Olympus Optical, Tokyo, Japan) and the bacterial count in 1mL was calculated.

*DNA extraction method and PCR conditions*

Samples of saliva and tracheal aspirate were diluted ten-fold with phosphate buffered saline (PBS). The 1,800 µL dilution was mixed with 200 µL of 30% SDS in a sterilized 15 mL tube. The mixture underwent 15-second sonication with an Astrason XL-2020 ultrasonic processor (Antrason) three times. Then, the solution was treated with an equal amount of TE buffer saturated phenol. After the centrifugation, the DNA in the supernatant was purified and concentrated with an Amicon ultra centrifugal filter 100K (Millipore, Bedford, MA, U.S.A.).

The bacterial 16S rRNA genes were amplified according to the previous reports [1,2]. The 25 µL of PCR reaction mixtures containing the universal primers set [3] (E341F; 5’-CCTACGGGAGGCAGCAG-3’ and E907R; 5’-CCGTCAATTCMTTTRAGTTT-3’) and AmpliTaq Gold DNA polymerase LD (Applied Biosystems; Foster City, CA) were prepared. Then the reaction mixtures were incubated at 96ºC for 5 min. This was followed by 30 cycles at 96ºC for 30 s, 53ºC for 30 s and 72ºC for 1 min and a final elongation step at 72ºC for 7 min.

*Clone library construction and nucleotide sequencing analysis*

This analysis was performed as described previously [1,2]. Cloning and sequencing analysis was performed with PCR positive (30 cycles) samples only. Regarding the samples with no obvious band on 2% agarose gel electrophoresis analysis, it was judged to be PCR negative and did not attempt cloning. The PCR products were cloned into *Escherichia coli* TOP10 cells using a TOPO TA cloning kit (Invitrogen; Carlsbad, CA) according to the manufacturer’s instructions. A total of 96 colonies were randomly selected from each clone library for sequencing analysis. The partial fragments of the cloning vectors (pCR II) containing inserted PCR products were amplified with AmpliTaq Gold 360 DNA polymerase and a primer set (M13Forward; 5’-GTAAAACGACGGCCAG-3’ and M13Reverse; 5’-CAGGAAACAGCTATGAC-3’). After the primers and deoxyribonucleotide triphosphate were eliminated from the PCR mixture with an ExoSAP-IT (GE Health care UK Ltd.; England, UK) according to the manufacturer’s instructions, an aliquot (1µL) was used for the sequencing reaction. The sequencing reactions were accomplished with a primer ‘‘M13Forward’’ and the BigDye Terminator Cycle Sequencing Kit v3.1 (Applied Biosystems). The nucleic acid sequences were determined on a 3130*xl* Genetic Analyzer (Applied Biosystems).

Data analysis was performed using the software ‘Sequencing Analysis V.5.2’ (Applied Biosystems). The sequences remaining after the quality check (length of read >500 nucleotides and average quality value of 20 bases ≥ 20) and only the precisely read sequences of universal primer E341F and E907R were selected. In addition, the raw data was checked and the sequences including ambiguous bases between the primer sequences were excluded. The remaining highly accurate sequences were trimmed to remove primer and vector sequences and roughly 550 base pairs were classified into the level of genus with RDP (ribosomal data project) classifier (confidence threshold 90% cut-off value) [4]. The sequences less than 90% value were deemed unclassified. Genera with proportions less than 0.1% of the total number of clones were titled ‘others’. Moreover, in-house database (Appendix S2) containing 5878 16S rRNA gene sequences of type strains using the BLAST (basic local alignment search tool) algorithm was used for more detail analysis at the level of species. All sequences were submitted to public database (DDBJ, Accession number: LC350341-LC360093).

**References**

1. Kawanami T, Fukuda K, Yatera K, Kido M, Mukae H, Taniguchi H. A higher significance of anaerobes: the clone library analysis of bacterial pleurisy. *Chest* 2011;139(3):600–608.
2. Kawanami T, Fukuda K, Yatera K, et al. Severe pneumonia with Leptotrichia sp. detected predominantly in bronchoalveolar lavage fluid by use of 16S rRNA gene sequencing analysis. *J Clin Microbiol* 2009;47(2):496–498.
3. Lane DJ. 16S/23S rRNA sequencing. In: Strackebraundt E, Goodfellow M, eds. *Nucleic acid techniques in bacterial systematics*. New York, NY: John Wiley & Sons Ltd. 1991;115–175.
4. Wang Q, Garrity GM, Tiedje JM, Cole JR. Naïve Bayesian Classifier for Rapid Assignment of rRNA Sequences into the New Bacterial Taxonomy. Appl Environ Microbiol. 2007;73:5261-5267.

**Additional figure legends**

**Figure S1:** Proportions of microbiota in saliva and tracheal aspirate (individual cases). Obligate anaerobes are indicated as orange background (Species of *Actinomyces*, *Campylobacter*, and *Treponema* detected in this study were obligate anaerobes). Aerobes and facultative anaerobes are indicated as white background. Others is defined as genus with proportions less than 0.1% of the total number of clones. The sequences less than 90% value were deemed unclassified. (A): samples collected within 2 hours after intubation, (B): samples collected just before administration of antibiotics, (C): samples collected 48-72 hours after the administration of antibiotics.

**Figure S2:** Unsupervised hierarchical clustering of genus for samples of saliva and tracheal aspirate. Obligate anaerobes are indicated as orange background (Species of *Actinomyces*, *Campylobacter*, and *Treponema* detected in this study were obligate anaerobes). Aerobes and facultative anaerobes are indicated as white background. Samples with early recruitment group (day 0) are indicated as yellow background. Samples with late recruitment group (day 1 or more) are indicated as blue background. Others is defined as genus with proportions less than 0.1% of the total number of clones. The sequences less than 90% value were deemed unclassified. (A): samples collected within 2 hours after intubation, (B): samples collected just before administration of antibiotics, (C): samples collected 48-72 hours after the administration of antibiotics.

**Figure S3:**  Eigenvectors of PC 1 and 2 by bacterial genera. PC 1: Principal Component 1, PC 2: Principal Component 2.

**Figure S4:**  Dynamics of microbiota in saliva and tracheal aspirate.

(A): samples collected within 2 hours after intubation, (B): samples collected just before administration of antibiotics, (C): samples collected 48-72 hours after the administration of antibiotics.
