# Supplementary figures and images for: Dynamics of microbiota during mechanical ventilation in aspiration pneumonia
Source: BMC Pulm Med. 2019 Dec 23;19:260. doi: 10.1186/s12890-019-1021-5 (PMC6929358; doi:10.1186/s12890-019-1021-5)

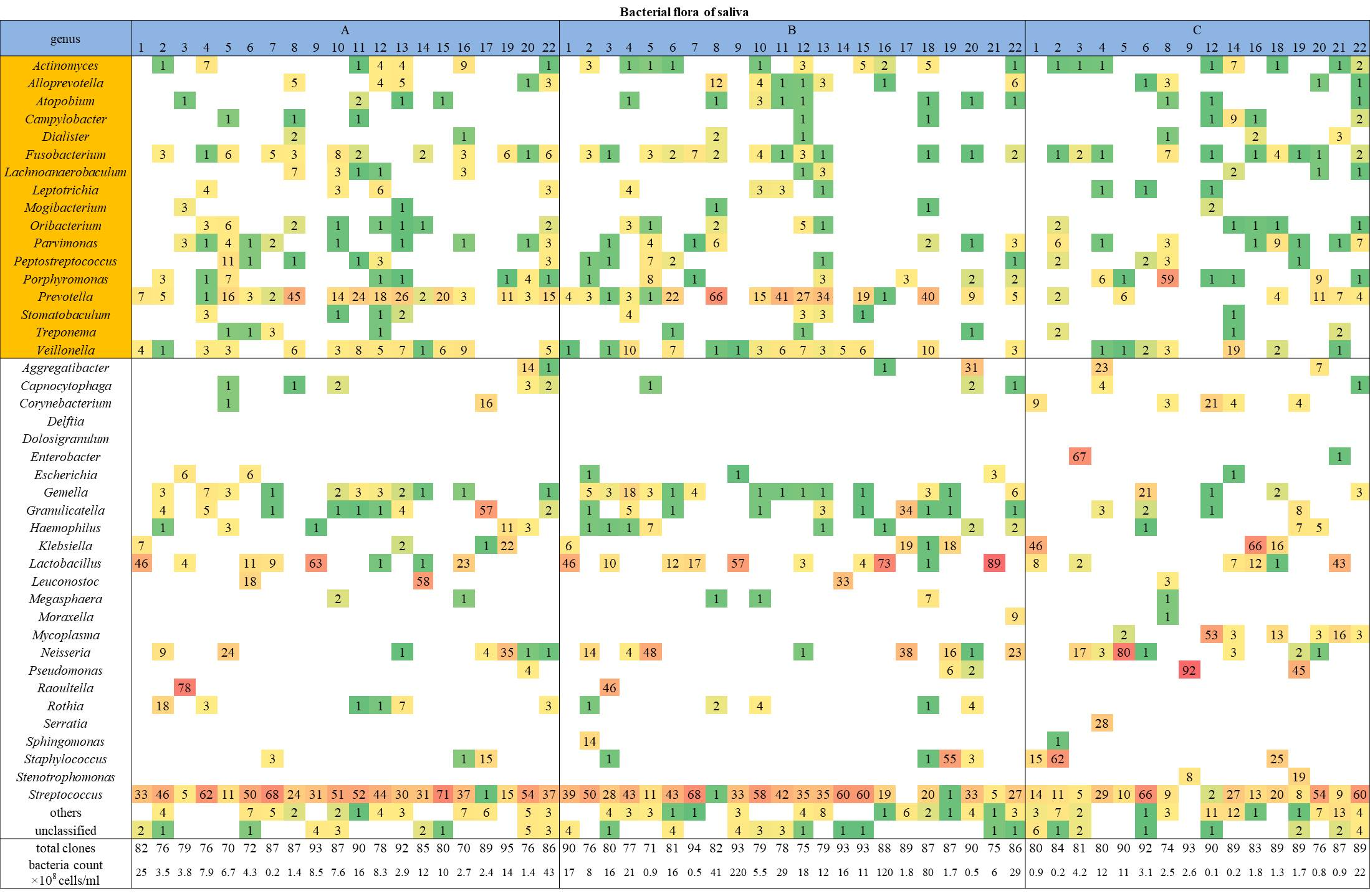

Supplement: Supplementary file 3 — Additional file 3: Figure S1–1, S1–2. Proportions of bacterial flora in saliva and tracheal aspirate (individual cases). [file 12890_2019_1021_MOESM3_ESM.zip › S1-1 FigR2.tif]

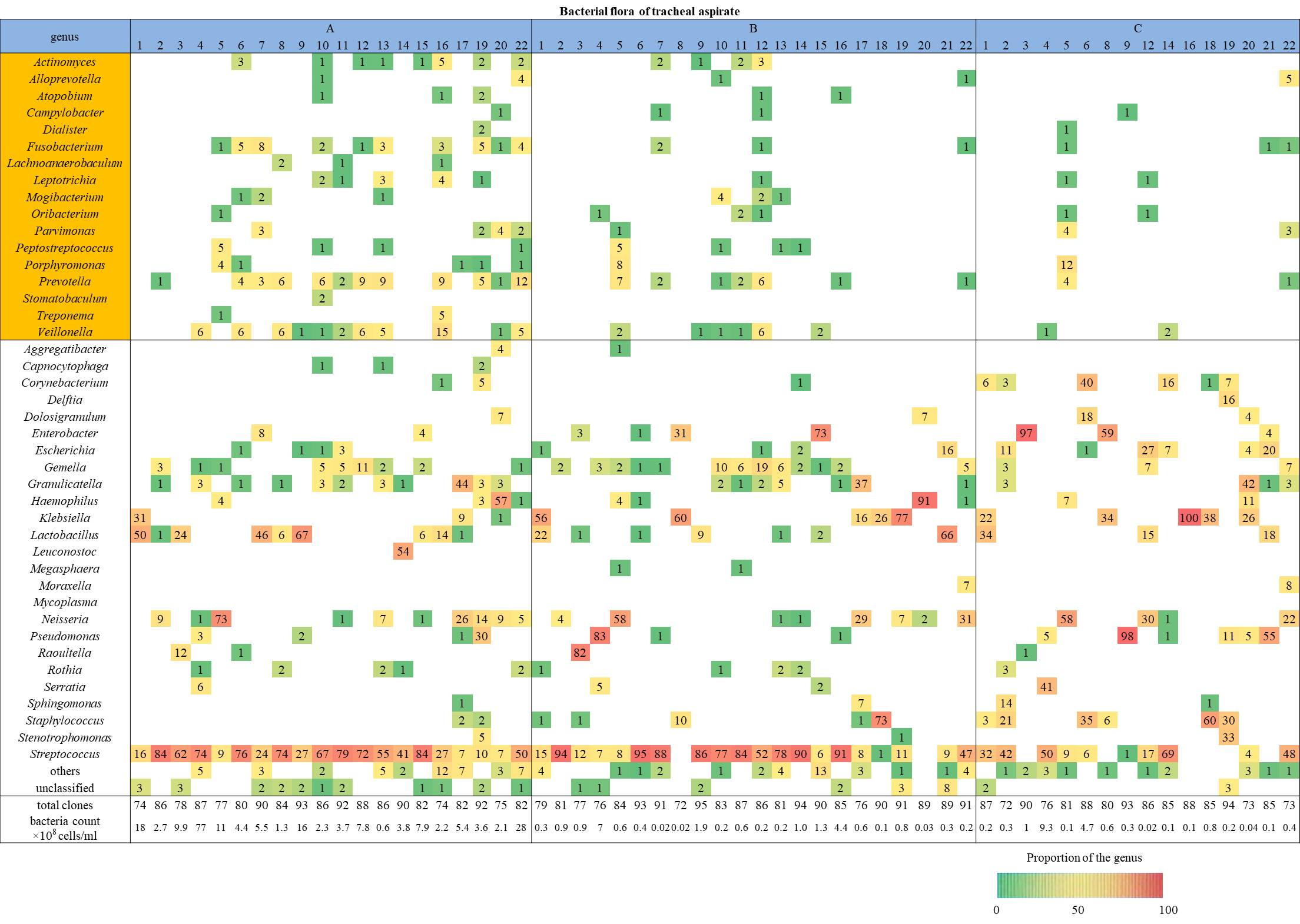

Supplement: Supplementary file 3 — Additional file 3: Figure S1–1, S1–2. Proportions of bacterial flora in saliva and tracheal aspirate (individual cases). [file 12890_2019_1021_MOESM3_ESM.zip › S1-2 FigR2.tif]

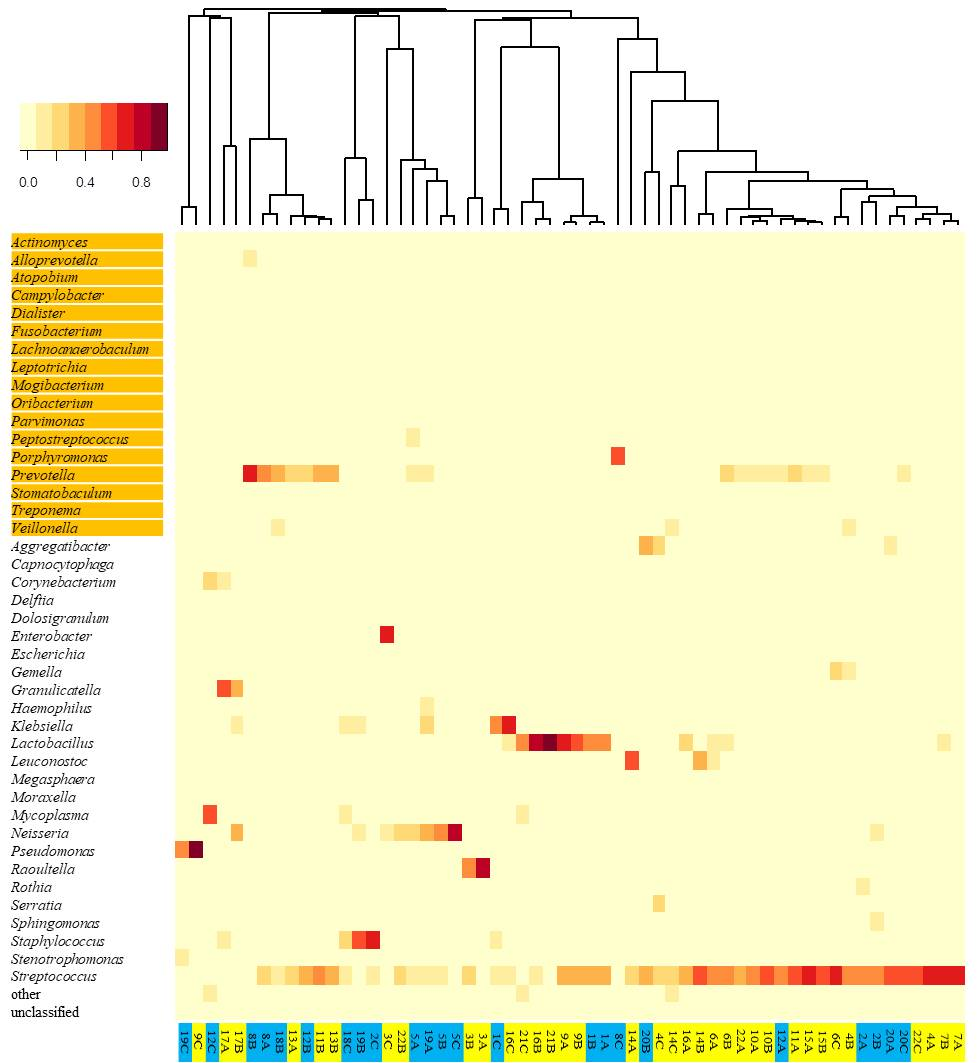

Supplement: Supplementary file 4 — Additional file 4: Figure S2–1, S2–2. Unsupervised hierarchical clustering of genus for samples of saliva and tracheal aspirate. [file 12890_2019_1021_MOESM4_ESM.zip › S2-1 FigR2.tif]

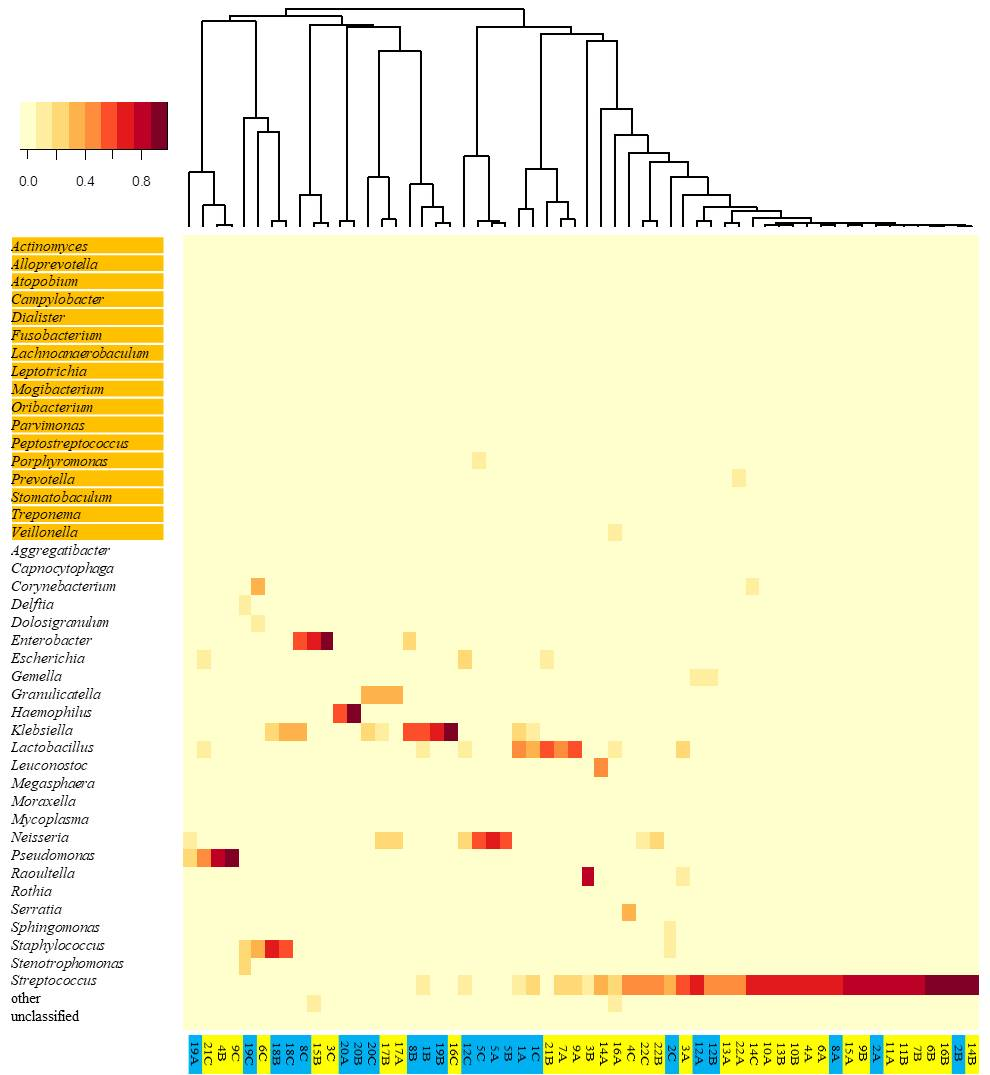

Supplement: Supplementary file 4 — Additional file 4: Figure S2–1, S2–2. Unsupervised hierarchical clustering of genus for samples of saliva and tracheal aspirate. [file 12890_2019_1021_MOESM4_ESM.zip › S2-2 FigR2.tif]

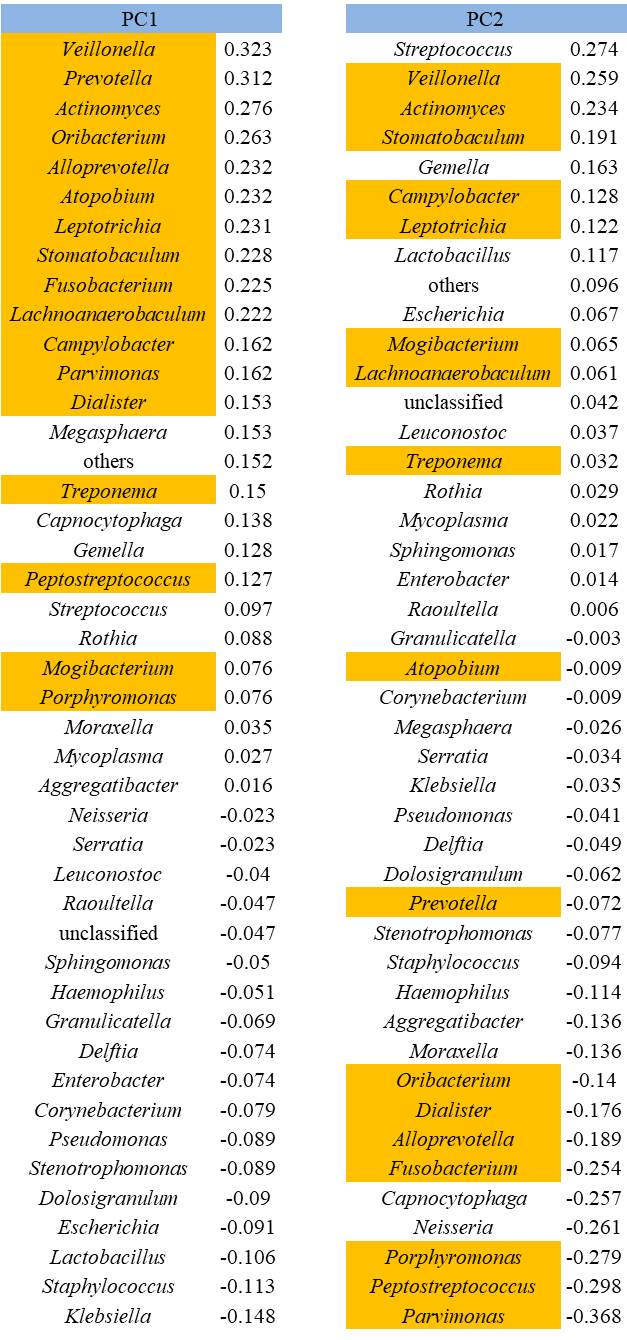

Supplement: Supplementary file 6 — Additional file 6: Figure S3. Eigenvectors of PC 1 and 2 by bacterial genera. [file 12890_2019_1021_MOESM6_ESM.tif]

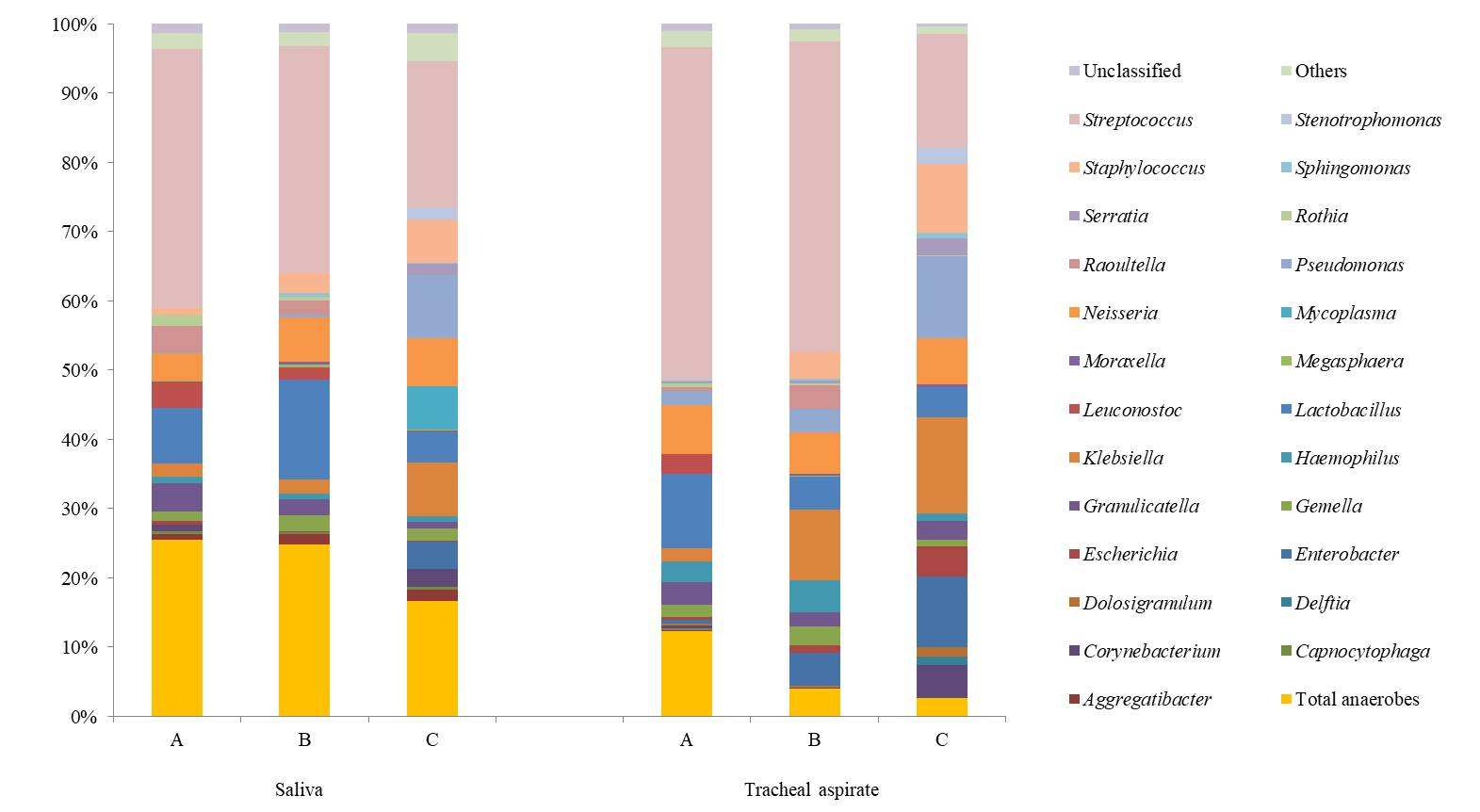

Supplement: Supplementary file 7 — Additional file 7: Figure S4. Dynamics of microbiota in saliva and tracheal aspirate. [file 12890_2019_1021_MOESM7_ESM.tif]
