# Supplementary material for: Dynamics of microbiota during mechanical ventilation in aspiration pneumonia
Source: BMC Pulm Med. 2019 Dec 23;19:260. doi: 10.1186/s12890-019-1021-5 (PMC6929358; doi:10.1186/s12890-019-1021-5)
Supplement: Supplementary file 5 — Additional file 5: Table S1. Predominant Phylotypes Detected by Clone Library Method in 22 Subjects. [file 12890_2019_1021_MOESM5_ESM.docx]

| **Table S1. Predominant phylotypes detected by clone library method in 22 subjects** | | | | |
| --- | --- | --- | --- | --- |
| Sample | Order | Predominant Phylotype | | |
|  |  | (A) | (B) | (C) |
| Saliva | first | *Streptococcus* | *Streptococcus* | *Streptococcus* |
|  | second | *Prevotella* | *Lactobacillus* | *Pseudomonas* |
|  | third | *Lactobacillus* | *Prevotella* | *Klebsiella* |
|  |  |  |  |  |
| Tracheal aspirate | first | *Streptococcus* | *Streptococcus* | *Streptococcus* |
|  | second | *Lactobacillus* | *Klebsiella* | *Klebsiella* |
|  | third | *Neisseria* | *Neisseria* | *Pseudomonas* |
|  | | | | |
| (A)=samples collected within 2 hours after intubation, (B)=samples collected just before administration of antibiotics, (C)=samples collected 48-72 h after the administration of antibiotics | | | | |
